# Purification, characterization and crystallization of the F-ATPase from *Paracoccus denitrificans*

**DOI:** 10.1098/rsob.150119

**Published:** 2015-09-30

**Authors:** Edgar Morales-Rios, Ian N. Watt, Qifeng Zhang, Shujing Ding, Ian M. Fearnley, Martin G. Montgomery, Michael J. O. Wakelam, John E. Walker

**Affiliations:** 1 The Medical Research Council Mitochondrial Biology Unit, Cambridge Biomedical Campus, Hills Road, Cambridge CB2 0XY, UK; 2 The Babraham Institute, Cambridge CB22 3AT, UK

**Keywords:** α-proteobacteria, *Paracoccus denitrificans*, F-ATPase, subunits, cardiolipin, crystallization

## Abstract

The structures of F-ATPases have been determined predominantly with mitochondrial enzymes, but hitherto no F-ATPase has been crystallized intact. A high-resolution model of the bovine enzyme built up from separate sub-structures determined by X-ray crystallography contains about 85% of the entire complex, but it lacks a crucial region that provides a transmembrane proton pathway involved in the generation of the rotary mechanism that drives the synthesis of ATP. Here the isolation, characterization and crystallization of an integral F-ATPase complex from the α-proteobacterium *Paracoccus denitrificans* are described. Unlike many eubacterial F-ATPases, which can both synthesize and hydrolyse ATP, the *P. denitrificans* enzyme can only carry out the synthetic reaction. The mechanism of inhibition of its ATP hydrolytic activity involves a ζ inhibitor protein, which binds to the catalytic F_1_-domain of the enzyme. The complex that has been crystallized, and the crystals themselves, contain the nine core proteins of the complete F-ATPase complex plus the ζ inhibitor protein. The formation of crystals depends upon the presence of bound bacterial cardiolipin and phospholipid molecules; when they were removed, the complex failed to crystallize. The experiments open the way to an atomic structure of an F-ATPase complex.

## 1. Introduction

Our current knowledge of the rotary mechanism of ATP synthase is based largely on the analysis of the structure of the enzyme from the inner membranes of mitochondria [1–4] coupled with ‘single-molecule’ observations of the enzyme's rotary mechanism conducted almost entirely on enzymes from eubacteria [5]. The F-ATPase from bovine mitochondria has been characterized by structural analysis most extensively, and detailed structures representing almost all of its constituent domains have been described [1–4]. They include over 25 structures of the globular membrane extrinsic F_1_-catalytic domain associated with various substrates, substrate analogues and inhibitors; the membrane extrinsic region of the peripheral stalk, a key component of the enzyme's stator and its mode of interaction with the F_1_-domain; and the structure of the membrane bound c-ring, a central component of the enzyme's rotor attached to the rest of the rotor, the central stalk component in the F_1_-domain [1–4]. These high-resolution component structures have been assembled into a mosaic high-resolution structure representing about 85% of the mitochondrial enzyme, within the constraints of a low-resolution overall structure of the monomeric bovine complex determined by cryo-electron microscopy [1,6]. The main missing element is the membrane bound segment of the stator including subunit a, which interacts with the rotating c-ring. Together the c-ring and the a-subunit provide the pathway for protons to cross the membrane in which the enzyme operates. Proton translocation through the membrane is an essential element in the generation of the rotation of the enzyme's rotor, driven by the transmembrane proton motive force produced by respiration or photosynthesis. If the mechanism of the generation of rotation from the proton motive force is to be understood, a detailed structure of this region of the complex is paramount. An initial view of this region of the enzyme has been provided by a cryo-electron microscopy analysis of the F-ATPase from *Polytomella* [7].

In contrast to the extensive structural studies conducted on the mitochondrial enzyme, rather few studies have been carried out of the structure of the F-ATPases from eubacteria. Their subunit compositions are somewhat simpler than those of mitochondrial enzymes [8–10]. They contain the same or analogous core subunits that constitute the catalytic domain, the rotor and the stator of mitochondrial enzymes, but they lack the six or more supernumerary membrane subunits found in the mitochondrial enzyme, that, as far as is known, play no role in the catalytic activity of the complex. Structures have been described of the F_1_-domains of the enzymes from *Escherichia coli* [11,12], *Caldalkalibacillus thermarum* [13] and *Bacillus* PS3 [14], of the α_3_β_3_-subcomplex also from *Bacillus* PS3 [15] and of isolated c-rings from the rotors of several species [16–20]. There is also fragmentary structural information concerning the peripheral stalk region of the F-ATPase from *E. coli*, describing the N-terminal domain of the δ-subunit and its mode of interaction with the N-terminal region of an α-subunit [21], and segments of the b-subunit [22–24].

Over the years, many attempts have been made to crystallize intact F-ATPases mainly from mitochondrial sources, as a prelude to determining their structures by X-ray crystallography, but with no success, until the work described here on the F-ATPase from the *α*-proteobacterium *Paracoccus denitrificans*. This organism has been proposed to have common ancestry with mitochondria and its respiratory chain has many similarities with mitochondrial respiratory chains [25]. Its F-ATPase is a complex of the nine core subunits. An inhibitor protein known as *ζ* binds to the catalytic domain to prevent the enzyme from hydrolysing ATP [26], and it is bound to the enzyme that has been crystallized, as described below.

## 2. Material and Methods

### 2.1. Analytical methods

Protein concentrations were measured by the bicinchoninic method (Pierce). The ATP hydrolase activity of the F-ATPase was measured by coupling the activity to the oxidation of NADH monitored at 340 nm [27]. The subunit composition of the purified F-ATPase complex was analysed by SDS-PAGE in 12–22% polyacrylamide gradient gels. Proteins were stained with 0.2% Coomassie blue dye or with silver. Samples of the purified enzyme were analysed by blue native PAGE on Bis-Tris Native PAGE 3–12% acrylamide gradient gels (Life Technologies).

### 2.2. Isolation of bacterial membranes

Cells of *P. denitrificans* (strain PD1222; Rif^r^, Spe^r^; enhanced conjugation frequencies; host-specific modification, m^+^) were grown as described elsewhere [28]. A suspension of the cells (200 g) in buffer (2 l) consisting of 10 mM Tris-HCl, pH 7.4, 0.5 M sucrose and 5 mM EDTA was digested for 3 h at room temperature with lysozyme (1 g) and then centrifuged (15 180*g*, 20 min, 4°C). The pellet was resuspended in buffer containing 10 mM Tris-acetate, pH 7.4, 0.1 mM ATP and Complete EDTA-free protease inhibitors (Roche; 1 tablet/100 ml). The suspension was treated for 30 min with DNAse I (10 mg; 2900 U) in the presence of 5 mM MgCl_2_ (final concentration) and centrifuged (31 916*g*, 50 min, 4°C). The upper bright red-brown layer of the pellet was resuspensed in buffer (1 l) containing 10 mM Tris-HCl, pH 7.4 and 1 mM MgSO_4_, centrifuged (31 916*g*, 50 min, 4°C), and resuspended again in buffer containing 10 mM Tris-acetate, pH 7.5, 10% glycerol, 1 mM ATP, 1 mM MgCl_2_ and Complete EDTA-free protease inhibitor tablets (1 tablet/100 ml; protein concentration 20–30 mg ml^−1^). The final suspension of membranes (*ca* 120 ml) was divided in 30 ml portions and stored at −80°C.

### 2.3. Purification of the F-ATPase

Membranes from *P. denitrificans* (30 ml; 30 mg of protein ml^−1^) were diluted to a protein concentration of 10 mg ml^−1^ in buffer containing 50 mM bis-Tris, pH 7.4, and 750 mM aminocaproic acid. Undecyl-β-d-maltoside was added from a 10% solution to give a detergent : protein ratio of 1 : 1 (w:w). The suspension was centrifuged (224 468*g*, 4°C, 35 min), and the supernatant was fractionated by chromatography as follows. Nickel ions were displaced from two HisTrap HP columns (each 5 ml; GE Healthcare) by washing first with three column volumes each of 100 mM EDTA, and then 0.1 M CuCl_2_. These columns are referred to as HisTrap HP (Cu) columns. They were connected in series and were followed by a HisTrap (Ni) column (5 ml; GE Healthcare) and two HiTrap Q HP columns (each 5 ml; GE Healthcare). This train of columns was equilibrated in buffer consisting of 50 mM Tris-HCl, pH 7.4, 1 mM MgCl_2_, 10% glycerol, 0.5 mM ATP, 0.05% undecyl-β-d-maltoside and Complete EDTA-free protease inhibitor tablets (1 tablet : 100 ml), and then the sample of solubilized membranes from *P. denitrificans* was applied. The columns were washed with buffer (150 ml), and then the HisTrap HP (Cu) and (Ni) columns were removed. The two remaining Q HiTrap columns were eluted with a step gradient generated by mixing the column buffer (buffer A) with increasing amounts of the same buffer containing 0.5 M sodium chloride (buffer B). The steps were 10, 20, 30, 37, 42, 47, 55 and 100% of buffer B in buffer A. The F-ATPase eluted in two separate peaks at 47% and 55% buffer B (corresponding to a salt concentration of 230 and 260 mM sodium chloride, respectively). They are referred to as F-ATPases I and II, respectively. The fractions collected from these columns were analysed by SDS-PAGE, and those containing the purest enzyme from each peak were pooled separately (total volume of each 20 ml), and concentrated by centrifugation (2939*g*; 5°C) through a Vivaspin 20 ultrafiltration concentrator (molecular weight cut off 100 kDa; Sartorius Stedim Biotech). The concentrates (500 µl; protein concentration 30 mg ml^−1^) were applied separately to a Superose 6 gel filtration column (10 × 300 mm; GE Healthcare) equilibrated in buffer containing 50 mM Tris-HCl, pH 7.4, 1 mM MgCl_2_, 10% glycerol, 0.5 mM ATP, 0.05% undecyl-β-d-maltoside (w/v) and Complete EDTA-free protease inhibitor tablets (1 tablet : 100 ml). The flow rate of the buffer was 0.5 ml min^−1^, and the fractions containing the purest F-ATPases I and II were identified by SDS-PAGE, pooled (total volume 2 ml) and concentrated by ultrafiltration as above (final volume of 200 µl; protein concentration 15 mg ml^−1^).

### 2.4. Crystallization of the F-ATPase from *Paracoccus denitrificans*

F-ATPases I and II (each 15 mg ml^−1^) in 50 mM Tris-HCl, pH 7.4, 1 mM MgCl_2_, 10% glycerol, 0.5 mM ATP, 0.05% undecyl-β-d-maltoside and Complete EDTA-free protease inhibitor tablets (1 tablet : 100 ml) were mixed with an equal volume of buffer, consisting of 50 mM Tris-HCl, pH 8.0, 70–100 mM MgCl_2_ and 18–20% polyethylene glycol 4000. Sitting drops (1 µl) were formed in 96-well MRC plates (Swissci, Zug, Switzerland) for growth of crystals by vapour diffusion. Crystals formed from F-ATPase I only, and they were grown for 20 days at 25°C. Twenty crystals were harvested individually, each on a micromount. Each crystal was dipped sequentially, for 10 s for each dip, into three portions of buffer with the same composition as the mother liquor. These crystals were pooled and their protein contents were analysed by SDS-PAGE. Other crystals were harvested with micromounts (Mitegen, Ithaca, NY, USA) and cryoprotected by transfer sequentially through five drops of buffer, containing 50 mM Tris-HCl, pH 8.0, 50 mM MgCl_2_, 0.1% (w:v) undecyl-β-d-maltoside, 20% glycerol (v:v) and 19% (w:v) polyethylene glycol 4000. They were equilibrated in each drop for 1 min, flash-frozen in liquid nitrogen and stored at −80°C.

X-ray diffraction data were collected on an in-house Rigaku FR-E+ superbright X-ray source (Rigaku, Houston, TX, USA), at the Diamond Light Source, Harwell, Oxfordshire, UK and at the European Synchrotron Radiation Facility, Grenoble, France.

### 2.5. Analysis of lipids bound to F-ATPases I and II

The solvents employed for extraction and analysis of lipids were LC-MS grade (Fisher Scientific). Standard lipids were purchased from Avanti Polar Lipids (Alabaster, Alabama, USA). Samples of F-ATPases I and II from *P. denitrificans* (500 µl; 15 mg ml^−1^) were exchanged into a buffer consisting of 10 mM Na_2_HPO_4_, 1.8 mM KH_2_PO_4_, 137 mM NaCl, 2.7 mM KCl, 1 mM MgCl_2_, 0.5 mM ATP, 0.05% undecyl-β-d-maltoside and 1 Complete protease inhibitor tablet per 100 ml of buffer by passage of the enzyme through a column of Superose 6 (10 × 300 mm) equilibrated in the same buffer. Samples of the F-ATPase (100 µl; 15 mg ml^−1^) were diluted with 50% aqueous methanol (2.9 ml), and chloroform (3 ml), and a standard mixture of synthetic lipids containing C17-acyl groups rather than the even numbers of carbon atoms (predominantly C16 and C18) in the acyl groups of natural lipids (40 µl). This sample of lipid standard contained 17 : 0-cholesterol ester (CE; 400 ng), cholesterol-d7 (CH-d7; 1000 ng), 17 : 1/17 : 1/17 : 1-triacylglycerol (TG; 800 ng), 17 : 0/18 : 1-diacylglycerol (DG; 200 ng), 17 : 0-monoacylglycerol (MG; 100 ng), 17 : 0-free fatty acid (FFA; 400 ng), 17 : 0-fatty acyl coenzyme A (FaCoA; 100 ng), 17 : 0-fatty acyl carnitine (FaCN; 50 ng), 17 : 0/18 : 1-phosphatidic acid (PA; 50 ng), 17 : 0/18 : 1-phosphatidylcholine (PC; 400 ng), 17 : 0/18 : 1-phosphatidylethanolamine (PE; 200 ng), 17 : 0/18 : 1-phosphatidylglycerol (PG; 50 ng), 17 : 0/20 : 4-phosphatidylinositol (PI; 400 ng), 17 : 0/18 : 1-phosphatidylserine (PS; 200 ng), 14 : 0/14 : 0/14 : 0/14 : 0-cardiolipin (CL; 200 ng), C17-platelet-activating factor (PAF; 50 ng), C17-2-lysoplatelet-activating factor (LysoPAF; 50 ng), 17 : 0-2-lysophosphatidic acid (LPA; 50 ng), 17 : 0-2-lysophosphatidylcholine (LPC; 100 ng), 17 : 1-2-lysophosphatidylethanolamine (LPE; 100 ng), 17 : 12-lysophosphatidylglycerol (LPG; 50 ng), 17 : 1-2-lysophosphatidylinositol (LPI; 100 ng), 17 : 1-2-lysophosphatidylserine (LPS; 50 ng), C17-ceramide (Cer; 50 ng), C17-sphingosine (SG; 50 ng), 12 : 0-ceramide-1-phosphate (Cer1P; 50 ng), C17-sphingosine-1-phosphate (S1P; 50 ng), C17-sphingomyelin (SM; 400 ng), C17-sphingosine-1-phosphocholine (S1P; 50 ng) and C17-monosulfogalatosyl ceramide (Sul-Gal-Cer; 50 ng). This mixture was subjected to Folch extraction [29]. The lower phase was recovered, and the upper aqueous phase was re-extracted with chloroform : methanol : water; (2 : 1 : 1, by vol; 3 ml), the same composition as the lower phase. The combined lower phase extracts were evaporated *in vacuo* at 18°C and re-dissolved in chloroform (70 µl). Samples (7 µl) were analysed by LC/MS/MS with both positive and negative electrospray ionization. Lipids were fractionated on a normal phase silica gel column (2.1 × 150 mm, particle size 4 µm; MicroSolv Technology, Eatontown, NJ, USA) with a ternary gradient of solvents. The column was mounted in a Prominence high performance liquid chromatograph (Shimadzu). It was equilibrated with a 3 : 1 mixture of hexane and chloroform (v:v) and eluted with a gradient of solvent B consisting of dichloromethane : chloroform : methanol (45 : 45 : 10, by vol) containing 0.08% ethylamine (v:v), followed by a gradient from solvent B to solvent C, consisting of chloroform : methanol : acetonitrile : water (30 : 30 : 30 : 10, by vol) containing 0.12% ethylamine (v:v). The column eluent was supplemented with *ca* 10% (v/v) 20 mM ammonium formate in 50% aqueous methanol during the gradient from solvents B to C. It was directed into a Thermo Orbitrap Elite mass spectrometer (Thermo Fisher) operated in single ion monitoring scan mode as described previously [30,31]. Selected ions were fragmented by collision-induced dissociation in the ion trap. The naturally occurring lipids originating from the samples of F-ATPase were identified by reference to the synthetic standards, and quantitated from the peak areas in the ion current traces.

### 2.6. Mass spectrometric analysis of subunits of F-ATPase I

Stained bands containing the subunits of the *P. denitrificans* F-ATPase I were analysed by mass fingerprinting and tandem MS analysis of tryptic peptides in a 4800+ MALDI-TOF–TOF mass spectrometer (Applied Biosystems, Foster City, CA, USA) and a Thermo Orbitrap XL electron transfer dissociation instrument (Thermo Scientific, Waltham, MA, USA). In the case of the a-subunit, additional experiments were conducted on a chymotrypic digest. The masses of peptides and their partial sequences obtained by collision-induced dissociation of peptide ions were compared via MASCOT (Matrix Sciences, London, UK) with a protein sequence database of the National Center for Biotechnology Information and against a local protein sequence database [32]. The subunits of F-ATPase I from *P. denitrificans* were fractionated by reverse-phase chromatography as described before [33], and the eluate was introduced ‘online’ via an electrospray interface into a Q-Trap 4000 mass spectrometer (ABSciex). The instrument was operated in MS mode and was calibrated with a mixture of myoglobin and trypsinogen [33]. Molecular masses were calculated with MassLynx (Waters) and Bioanalyst (ABSciex).

## 3. Results

### 3.1. Purification of the F-ATPase from *Paracoccus denitrificans*

**Figure 1.**
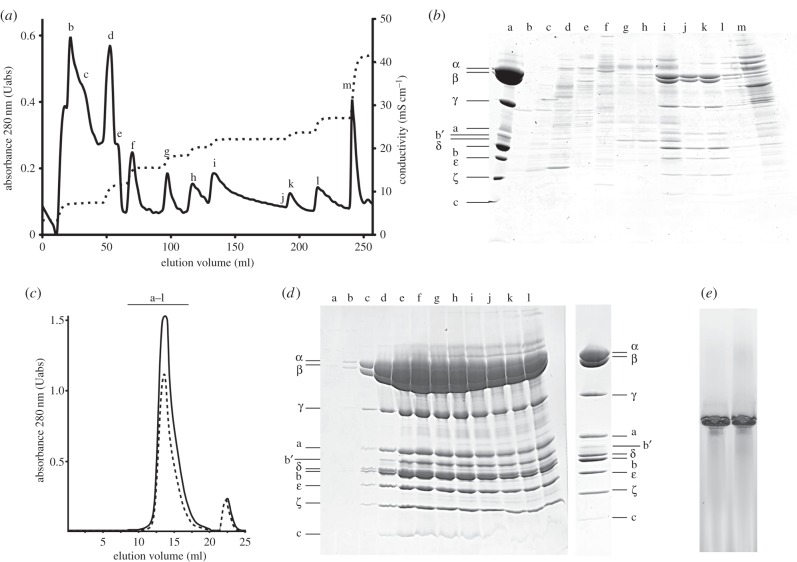
Purification and characterization of the F-ATPase from *P. denitrificans*. (*a*) Anion exchange chromatographic fractionation on two tandem Q HiTrap HP columns of initial membrane extract produced in the presence of undecyl-β-d-maltoside. The proteins were eluted with a step gradient of increasing concentrations of sodium chloride monitored via the conductivity of the eluent (dotted line). The absorbance of the eluate was monitored at 280 nm (solid line). (*b*) Analysis by SDS-PAGE of proteins in peaks b–m in (*a*). Lane a is a marker of partially purified F-ATPase from *P. denitrificans*. The likely positions of subunits of the enzyme are indicated on the left. (*c*) Gel filtration of partially purified *P. denitrificans* F-ATPase from peaks i (solid lane, F-ATPase I) and peak k (dashed line, F-ATPase II) from (*a*). The absorbance of the eluate was monitored at 280 nm and fractions of 0.5 ml were collected. (*d*) Analysis by SDS-PAGE of fractions a–l from the solid line sample of (*c*); on the right is shown 10 µg of the pooled and concentrated fractions c–e. The positions of subunits of the enzyme as determined by mass mapping of tryptic peptides are indicated on the left for the fractions and on the right for the final F-ATPase. (*e*) Analysis by BN-PAGE of the purified F-ATPase I on the left and F-ATPase II on the right.

The F-ATPase was extracted from cellular membranes of *P. denitrificans* with undecyl-β-d-maltoside. Initially, attempts were made to bind the enzyme to a HisTrap column with bound nickel, and to a second HisTrap column, where the bound nickel had been displaced by cupric ions. Previously, a similar approach had been used successfully in the purification of the F-ATPase from the bacterium *Caldoalkalibacillus thermarum* strain TA2 [34]. However, the *P. denitrificans* F-ATPase did not bind to either column, but several contaminants were removed, and so both columns were retained in the purification process. The material emerging from these columns was fractioned by chromatography on a strong anion exchanger provided by a Q HiTrap column, which was developed with a step gradient of increasing salt concentration. The F-ATPase eluted in two separate but consecutive peaks (F-ATPases I and II, respectively; peaks l and k in figure 1*a* and *b*). Subsequently, F-ATPases I and II were treated separately. They were both purified further by gel filtration chromatography (figure 1*c*). The yields of F-ATPase I and of F-ATPase II were 3 mg and 2 mg, respectively, from 25 g of wet cells. Analysis on SDS-PAGE gels demonstrated that both F-ATPases I and II contained the nine constituent subunits of the enzyme (α, β, γ, δ, ε, b, b’, a and c) plus the ζ inhibitor protein (figure 1*d*), and after concentration on a membrane, which removed many of the minor contaminants, the preparations were free from any significant contaminants (exemplified by F-ATPase I in figure 1*d*, extreme right lane). Both F-ATPases I and II gave a single band on BN-PAGE gels (figure 1*e*).

### 3.2. Crystallization of F-ATPase I

**Figure 2.**
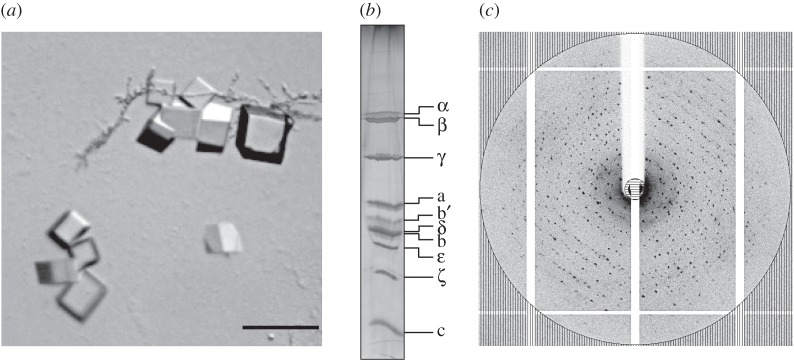
Crystals of F-ATPase I from *P. denitrificans*. (*a*) Twenty-day-old crystals grown by vapour diffusion. The bar represents 100 µm; (*b*) analysis of washed crystals by SDS-PAGE. The gel was silver stained. The identities of subunits of the enzyme are indicated on the right; (*c*) X-ray diffraction pattern of the crystals of F-ATPase I. The circle corresponds to a resolution of 6.8 Å.

Samples of F-ATPases I and II were set up for crystallization under identical conditions. However, crystals formed from F-ATPase I only, and none was obtained from F-ATPase II. They grew to their maximum size in about 20 days. They formed cubes with approximately 50 µm sides (figure 2*a*), and the crystals contained all of the subunits of the enzyme, plus the ζ inhibitor protein (figure 2*b*). These crystals diffracted X-rays to a resolution of 6.8 Å (figure 2*c*).

### 3.3. Composition of lipids bound to F-ATPases I and II

**Figure 3.**
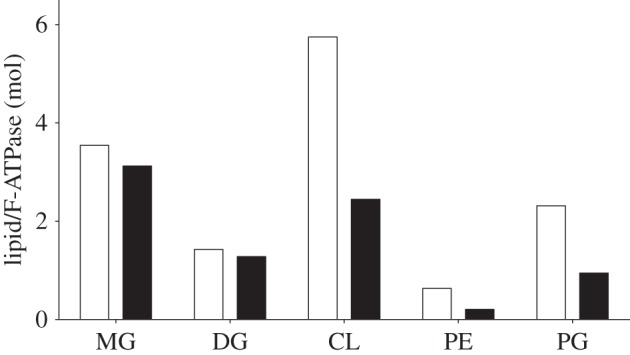
Analysis of lipids associated with F-ATPases I and II from *P. denitrificans*. The white and black histograms correspond to F-ATPases I and II, respectively. MG, monoacylglycerol; DG, diacylglycerol; CL, cardiolipin; PE, phosphatidylethanolamine; PG, phosphatidylglycerol. For details of the complete analysis see the electronic supplementary material, figure S1.

In order to investigate the differences in the ability of F-ATPases I and II to crystallize, the compositions of bound lipids were examined by quantitative measurement. These analyses demonstrated that both complexes had similar amounts of associated monoacylglycerol and diacylglycerol, but F-ATPase I had much more associated cardiolipin (almost six molecules per F-ATPase I as opposed to 2.5 molecules per F-ATPase II), and similarly F-ATPase I had about twice as much associated phosphatidylethanolamine and phosphatidylglycerol, albeit at significantly lower levels than cardiolipin (figure 3 and electronic supplementary material, figure S1). Therefore, the ability of the F-ATPase from *P. denitrificans* to form crystals appears to be dependent upon the retention of native lipids from the bacterial membrane.

### 3.4. Subunit composition of F-ATPase I

The subunit composition of F-ATPase I was analysed by mass spectrometry. By mass mapping and sequencing of the tryptic peptides from digests of the bands detected on SDS-PAGE gels, evidence was found for all nine of the expected constituent proteins of the F-ATPase, plus the *P. denitrificans* F-ATPase inhibitor protein, ζ (figure 1*d* and electronic supplementary material, table S1). In the case of the a-subunit, additional mass mapping of chymotryptic peptides was performed in order to increase the percentage coverage of the sequence of the protein from the constituent peptides (electronic supplementary material, table S1). The subunits were also fractionated by chromatography and their intact protein masses were measured (table 1). These experiments demonstrated that, with the exception of subunits δ, c, b and b’, the translational initiator formyl methionine residues had been removed post-translationally from the subunits and from the ζ inhibitor protein, and that in each instance, residue 2 provides the N-terminal residue of the mature protein. In the δ-subunit, either residues 1–3 or 1–26 were absent from the mature protein (table 1). In the case of the b-subunit residues 1–17 are removed, and for the b’-subunit no evidence was found for the presence of the full-length protein in the purified enzyme (mass 22388.3). However, based on reported interpretations of DNA sequences in databases, protein masses were observed that evidently correspond to truncated forms of the b’-subunit lacking residues 1–39, 1–42 and 1–43 (in relative abundances of 50, 35 and 15%, respectively). Two possibilities were considered, namely that the N-terminal region of the b’-subunit encoded in the corresponding gene had been removed by proteolysis, or that the initiator methionine residues for the gene for the b’-subunit had been mis-identified. In bacteria, the codon GTG can encode a formylmethionine translational initiator residue [10,35], and a plausible GTG initiation codon that gives a b’-subunit starting two residues before the longest version of the mature protein was identified (see the electronic supplementary material, figures S2 and S3). Thus, this codon may define the start of the coding region for the b’-subunit, which would therefore be 178 residues long, and according to this re-interpretation, two, five and six residues would be removed post-translationally from the N-terminus of subunit b’. However, this proposal is not yet established definitively. Mature b’-subunits have been defined in only two other F-ATPases, those from spinach chloroplasts [36–38] and *Rhodobacter capsulatus* [39]. Their sequences are aligned with the b’-subunit of *P. dentrificans* in the electronic supplementary material, figure S3.

**Table 1.** The masses of the subunits of the F-ATPase from *P. denitrificans* and of the inhibitor protein ζ.

subunit	observed (Da)	calculated (Da)^a^	Δ (Da)	modification
α	54 913.0	54 907.9	+5.1	−N-fMet
β	50 211.8	50 208.2	+3.6	−N-fMet
γ	31 469.5	31 467.9	+1.6	−N-fMet
δ	19 694.8	19 694.4	0	Δ1–3 or Δ1–26^b^
ε	15 692.9	15 692.7	0	−N-fMet
ζ	11 537.8	11 537.8	0	−N-fMet
a	26 593.7	26 592.8	0	−N-fMet
b	18 392.1	20 162.2	−1770.1	Δ1–17
b' (3–176)	18 454.9	18 454.9	0	Δ1–2^c^
b' (6–178)	18 255.7	18 255.7	0	Δ1–5
b' (7–178)	18 140.6	18 140.6	0	Δ1–6
c	7637.4	76 10.0	+27.4	+Nα-formyl

^a^The N-formyl methionine translational initiator is not included in these calculated values.

^b^The N-terminal sequence of the *δ* subunit is Ala-Asn-Ser-Ala-, and the DNA sequence of the *Atp* operon in *P. denitrificans* has two possible translational initiator methionine codons in the 5′-region before the DNA sequence encoding this N-terminal sequence. Thus, the generation of the observed mature protein would require the removal of either residues 1–3 or 1–26 from the initial product of translation.

^c^For a discussion of the N-terminus of subunit b’, see the text.

The mass spectrometric analyses also confirmed the association of the inhibitor protein, ζ, with the enzyme complex. From the peak areas from the mass spectrometric ion traces it was estimated that the amount of the ζ-protein in the complex is approximately equivalent to that of the δ-subunit, consistent with one ζ-protein per F-ATPase complex.

## 4. Discussion

### 4.1. Subunit compositions of eubacterial F-ATPases

Among the F-ATPases, those from eubacterial sources have the simplest subunit compositions, and the enzyme from *E. coli*, for example, is an assembly of eight different proteins [8,10]. Five of them, subunits α, β, γ, δ and ε, form the F_1_-catalytic domain with the stoichiometry 3 : 3 : 1 : 1 : 1, respectively [9]. Subunits α and β are arranged in a spherical α_3_β_3_-domain where catalysis occurs. Subunits γ and ε constitute the central stalk that penetrates into the α_3_β_3_-domain along its central axis, and together with a ring of c-subunits in the membrane domain of the enzyme they constitute the rotor of the enzyme. The number of c-subunits in the ring differs among eubacterial enzymes from 9 to 15 [16–20,40]. The role of the rotor during ATP synthesis is to transmit energy provided by the transmembrane proton motive force into the catalytic sites, to allow ATP to form. The δ-subunit binds to the ‘top’ of the α_3_β_3_-domain, and interacts with two identical b-subunits, which form an elongated α-helical structure, known as the peripheral stalk [41], connecting the α_3_β_3_-domain to the single a-protein in the membrane domain of the enzyme. The ensemble of the α_3_β_3_-domain, the δ-subunit, the two b-subunits and the a-subunit constitute the enzyme's stator against which the rotor turns. Subunit a is in intimate contact with the rotating c-ring, and together they provide a transmembrane pathway for protons. The subunit composition of the enzyme from *P. denitrificans* differs from that of this simplest F-ATPase only insofar as the two identical b-subunits are replaced by two related but non-identical subunits b and b’, and the F-ATPases in purple non-sulfur bacteria [35], cyanobacteria [42,43] and the chloroplasts [37,44,45] have a similar subunit composition. The F-ATPases in mitochondria have a homologous or analogous set of these ‘core’ subunits that constitute the eubacterial enzymes, but in addition they have a number of supernumerary subunits in their membrane domains that appear to have no direct roles in the synthesis or hydrolysis of ATP [1].

### 4.2. Inhibition of ATP hydrolysis

The F-ATPases in some eubacteria, for example *E. coli*, are capable of both synthesizing and hydrolysing ATP [46]. Under aerobic conditions, they use the transmembrane proton motive force generated by respiration to drive the synthesis of ATP from ADP and phosphate, and during anaerobiosis they reverse their action and hydrolyse ATP produced by glycolysis to generate a proton motive force. By contrast, in other eubacteria such as *C. thermarum* [47], *Mycobacterium smegmatis* [48] and *P. denitrificans* [26] the F-ATPase can only synthesize ATP, and the hydrolytic action is inhibited. The mechanism (or mechanisms) of inhibition is (are) poorly understood, but in *P. denitrificans* it involves the inhibitor protein ζ, which is conserved throughout α-proteobacteria, but not in other classes of eubacteria [26]. The ζ protein consists mainly of four α-helices in a down–up–down–up bundle (residues 19–42, 46–53, 66–77 and 81–103) [49]. Its N-terminal region from residues 1–18 is unstructured in solution, and the inhibitory activity of the protein lies within residues 1–14 [50]. It is bound to the preparation of the F-ATPase described here, and earlier studies suggest that it will be bound to the F_1_-domain in the vicinity of the C-terminal regions of the α- and β-subunits, but its precise mode of binding and mechanism of inhibition are not known.

### 4.3. Crystallization of intact F-ATPases

F-ATPases have been purified by affinity chromatography from the mitochondria of a wide range of species [51–53], and these enzyme preparations are available in sufficient quantities to sustain the exploration of a wide range of conditions that might lead to the formation of crystals suitable for X-ray crystallographic studies. However, despite extensive efforts, no such crystals have been obtained hitherto with the bovine enzyme and with fungal enzymes from two species. Thus, the successful crystallization of the enzyme from *P. denitrificans* may provide practical indications of why these experiments failed, and how future experiments might be conducted more effectively. The present experiments also suggest that identification of the key factors that led to success in the current experiments could help in the crystallization of F-ATPases from other bacterial species.

The first factor is that, according to the analytical criteria that were applied, the *P. denitrificans* enzyme appears to be free from other protein components of the bacterial membranes from which it originated. Only authentic subunits of the enzyme were detected in the final purified product (figure 1*d*, extreme right lane), and the enzyme complex was entirely monomeric (figure 1*e*). Similar comments can be made about the chemical purity of the subunit composition of the various mitochondrial F-ATPases that have been described. However, it is now apparent that some of the ‘supernumerary’ membrane subunits found in mitochondrial enzymes can be lost, wholly or partially, during purification, leading to inhomogeneity in subunit composition of the purified enzyme [53]. An additional complicating factor is that, in the inner membranes of mitochondria, the F-ATPases are organized in dimers [54] and larger oligomeric structures, and the purified enzymes, although predominantly monomeric, may aggregate forming mixtures of monomeric and oligomeric forms.

Another striking feature that has emerged from the current investigations is that the crystallized enzyme has substantial amounts of bound endogenous lipids, and especially cardiolipin, and when the enzyme was prepared with lower amounts of bound lipids, then that preparation failed to produce protein crystals. The requirement for the presence of cardiolipin in order for preparations of F-ATPases to retain their membrane-associated activities is well known [55–57], and the structural roles of bound cardiolipin molecules in other respiratory complexes are well established [58–61]. In the case of the F-ATPase, it is possible that in addition to a possible structural role in stabilizing the complex, cardiolipin has a functional role and that its net two negative charges contribute to the proton exit pathway in the interface between the c-ring and subunit a [62].

One concern is that in consequence of its structural asymmetry in the peripheral stalk, for example, coupled with the structural asymmetry of the F_1_-domain, F-ATPases will contain a population of different structural isomers, and the presence of these isomers will impede or prevent the formation of crystals. There is no doubt that these isomers exist, as cryo-electron microscopy investigations of F-ATPases are demonstrating. However, the bovine F_1_-ATPase in complex with the membrane extrinsic region of the peripheral stalk also is presumably a mixture of structural isomers depending on how the peripheral stalk is aligned with the asymmetric surface of the F_1_-domain. Yet, the complex was crystallized, and a unique structure was determined by X-ray analysis [63]. Therefore, it appears from this earlier and present work that in each case one particular structural isomer dominates, or is particularly amenable to formation of crystals. These crystals potentially provide a route to defining the molecular structure of the enzyme at atomic resolution. However, the structure of that isomer may be a low energy state, and the understanding of the details of how this intricate machine works may depend on defining other conformational states. Here, cryo-electron microscopy is likely to provide a solution as the analysis of F-ATPase dimer from *Polytomella* indicates [7]. However, the level of structural detail that can be attained at the moment by cryo-electron microscopy, with such a flexible structure as the F-ATPase, is unlikely to surpass what can be attained by X-ray analysis of suitably diffracting crystals.

In recent work, crystals grown under similar conditions to those described above have allowed the structure of the intact F-ATPase from *P. denitrificans* to be solved by X-ray crystallography to 4 Å resolution. This structural analysis has demonstrated that, as expected from figure 2*b*, all of the nine subunits of the enzyme plus the *ζ* inhibitor protein are present in the crystallized complex. There are, as in other F-ATPases, three copies of α- and β-subunits, and one copy of each of the γ-, δ-, ε- and a-subunits. The peripheral stalk contains one copy of each of the b- and b’-subunits. The stoichiometry of the c-ring in the rotor domain of the enzyme, which varies among bacterial species, is 12. In addition, there is one *ζ* inhibitor protein bound to the F_1_-domain [28].
